# A tumor suppressor enhancing module orchestrated by GATA4 denotes a therapeutic opportunity for GATA4 deficient HCC patients

**DOI:** 10.7150/thno.38060

**Published:** 2020-01-01

**Authors:** Feng Lu, Qian Zhou, Lu Liu, Guandi Zeng, Weimin Ci, Wanting Liu, Gong Zhang, Zhiyi Zhang, Peng Wang, Aiqun Zhang, Yunfei Gao, Lei Yu, Qingyu He, Liang Chen

**Affiliations:** 1 Key Laboratory of Functional Protein Research of Guangdong Higher Education, Institute of Life and Health Engineering, College of Life Science and Technology, Jinan University, Guangzhou 510632, China.; 2 Beijing Institute of Genomics, Chinese Academy of Sciences, Beijing 100101, China; 3 Beijing Ditan Hospital, Beijing 100015, China; 4 Department of Hepatobiliary Surgery, Beijing Tsinghua Changgung Hospital, Beijing 100084, China; 5 Zhuhai Precision Medical Center, Zhuhai People's Hospital (Zhuhai Hospital Affiliated with Jinan University), Jinan University, Zhuhai 519000, Guangdong, China; 6 The Biomedical Translational Research Institute, Jinan University Faculty of Medical Science, Jinan University, Guangzhou 510632, Guangdong, China; 7 Beijing Tongren Hospital, Capital Medical University, Beijing 100730, China

**Keywords:** GATA4, Hepatocellular carcinoma, Tumor suppressor gene, targeting therapy

## Abstract

**Rationale**: Effective targeting therapies are limited in Hepatocellular carcinoma (HCC) clinic. Characterization of tumor suppressor genes (TSGs) and elucidation their signaling cascades could shed light on new strategies for developing targeting therapies for HCC.

**Methods**: We checked genome-wide DNA copy number variation (CNV) of HCC samples, focusing on deleted genes for TSG candidates. Clinical data, in vitro and in vivo data were collected to validate the tumor suppressor functions.

**Results**: Focal deletion of GATA4 gene locus was the most prominent feature across all liver cancer samples. Ectopic expression of GATA4 resulted in senescence of HCC cell lines. Mechanistically, GATA4 exerted tumor suppressive role by orchestrating the assembly of a tumor suppressor enhancing module: GATA4 directly bound and potently inhibited the mRNA transcription activity of β-catenin; meanwhile, β-catenin was recruited by GATA4 to promoter regions and facilitated transcription of GATA4 target genes, which were TSGs per se. Expression of GATA4 was effective to shrink GATA4-deficient HCC tumors in vivo. We also showed that β-catenin inhibitor was capable of shrinking GATA4-deficient tumors.

**Conclusions**: Our study unveiled a previously unnoticed tumor suppressor enhancing module assembled by ectopically expressed GATA4 in HCC cells and denoted a therapeutic opportunity for GATA4 deficient HCC patients. Our study also presented an interesting case that an oncogenic transcription factor conditionally functioned as a tumor suppressor when recruited by a TSG transcription factor.

## Introduction

Liver cancer is the 6^th^ most common cancer and the 2^nd^ most common cause of cancer-related mortality worldwide 1, with HCC the most common primary liver malignancy in adults. HCC is an end result of chronic liver disease and is often associated with alcoholic hepatitis, nonalcoholic steatohepatitis, virus-related hepatitis, and cirrhosis 2. Due to high prevalence of HBV infection, HCC poses a severe health threat to Chinese and African population, especially in Sub-Saharan region 3, 4.

Despite of advances in multimodalities of treatment including immunotherapies 5, the prognosis for HCC patient remains a dismal 5-year overall survival of less than 18% 6. The difficulties in treating HCC patients stem from our limited understanding of the mechanism underlying tumorigenesis at molecular level, which has been a field under intensive research during the recent years. Several intracellular signaling pathways have been closely associated with HCC: p53 pathway, retinoblastoma (Rb) pathway, transforming growth factor-beta (TGF-β), and Wnt/β-catenin pathway 7. Except a few relatively high-frequency oncogenes among limited number of HCC patients, we and others have reported that a significant portion of HCC tumors are negative for typical driver oncogenes 8, 9, thus limiting our ability to develop targeting therapy for HCC patients. Not surprisingly, the importance of identification and characterization of TSGs involved in development of HCC is being increasingly recognized in the hope of identifying druggable targets 9.

GATA4 is a typical member of zinc finger transcription factor family consisting of GATA 1-6, featuring family-specific 2 N-terminal transcription activation domains (TAD), 2 central zinc finger domains (ZF), a nuclear localizing signal (NLS) immediately C-terminal to ZF2 and a C-terminal region (CTR)^10^.

GATA4 binds to consensus sequence, A/TGATAA/G 11, and regulates expression of numerous target genes in a highly dynamic manner at transcriptomic level by binding to their promoter region 12, both during organogenesis 13 and in response to environmental cues 14. This can be explained by dynamic post-translational modifications imposed on it, which enhance or suppress its activity, including phosphorylation 10, 15, acetylation 16, 17, methylation 18, and SUMOylation 19. Moreover, GATA4 has also been reported to function as a pioneer modifier that opens up a closed chromatin to facilitate binding of transcription factors including itself to the target sites 20. Critical roles have been reported for GATA4 in controlling cell fate and development of embryo and liver 21-25. Its dysregulation is implicated in tumor development and progress.

In the light of dearth of targetable oncogenes for HCC patients, we recently started to identify TSGs at whole genome level. Here we report GATA4 as a functionally important HCC TSG. Ectopically expressed GATA4 orchestrated the assembly of a tumor suppressor enhancing module in HCC cells by directly binding and inhibiting the transcription activity of β-catenin to transcribe canonical Wnt pathway target genes. In the meantime, β-catenin was recruited by GATA4 to promoter regions to facilitate the transcription of TSGs which are GATA4 transcription targets. GATA4 thus created a tumor suppressor enhancing module between GATA4 itself and β-catenin. In vivo experiments showed that ectopic expression of GATA4 or administration of β-catenin inhibitors shrank GATA4-deficient HCC tumors. Our study revealed a previously unnoticed tumor suppressor enhancing module and shed light on a therapeutic opportunity for GATA4 deficient HCC patients. Our work also presented an interesting case that a typical oncogenic transcription factor functioned as a TSG when recruited by another TSG transcription factor.

## Results

### GATA4 is a potent and clinically relevant tumor suppressor gene for HCC

**Figure 1:**
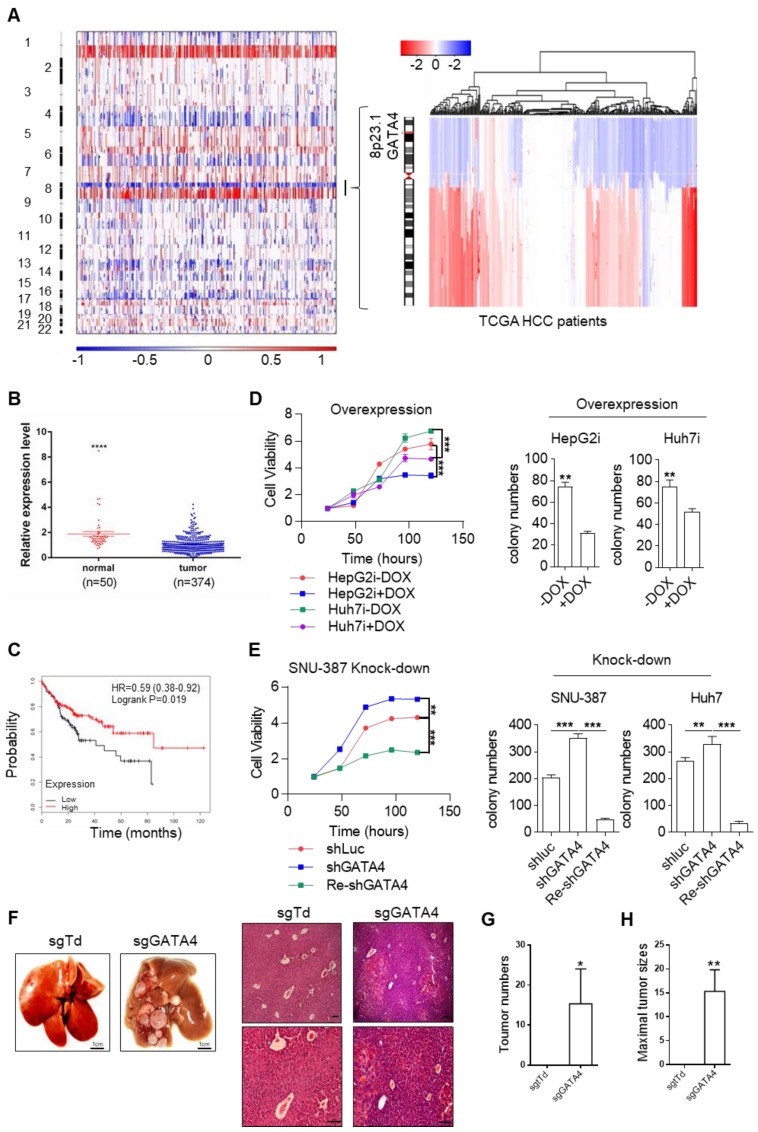
GATA4 is a potent and clinically relevant tumor suppressor gene for HCC. (a) Heatmap representation of genomic distribution and frequencies of CNV regions on autosomes in HCC samples. Segmental gains (red) and losses (blue) disturbed alone the chromosomes (Y-axis) was plotted against HCC patients (X-axis). CNV data of 358 HCC patients were retrieved from TCGA (left) Focused analysis of CNV of GATA4 region in chromosome 8 of the same batch of HCC patients (right). (b) GATA4 mRNA expression in liver cancer tissue and paratumoral tissue among the HCC patients from TCGA. (c) K-M survival of liver cancer patient (male, All stage, n=246). (d) **Left:** GATA4 expression negatively affected cell viability. HCC cell lines engineered for DOX-inducible expression of GATA4 (HepG2i and Huh7i) were treated w/o DOX (1 mg/mL) for 48 hours. Cell viability was assayed at indicated time. **Right:** GATA4 inhibited colony forming ability of HepG2 (left) and Huh7 (right) cells. (e) Effects of GATA4 knockdown on liver cancer cell lines. **Left:** Viability of SNU387 cells harboring shLuciferase (shLuc), shGATA4 or SNU387 cells harboring shGTA4 transfected with PC3.1-GATA4 plasmid (Re-shGATA4) were assayed with CCK8 reagents for the indicated time points.** Right:** Colony formation statistics of SNU387 (left) and Huh7 (right) cells. (f) GATA4 knockout enhanced HCC formation in vivo. Kras^lsl-G12D/+^ mice were infected with virus for simultaneous expression of Cre and CRISPR targeting GATA4 or TdTomato (serving as negative control) in hepatocytes. **Left:** Livers of the mice were shown at 6 months after lentivirus infection (scale bar, 1 cm),** Right:** H&E staining of liver sections of indicated genotypes (scale bars, 100 mm). (g) Quantification of liver surface tumor nodules number in indicated groups. (h) Maximal tumor sizes (diameters) were measure. Data are representative of three independent experiments, and were analyzed by unpaired t-test. Error bars denote SD *P < 0.05; **P < 0.01; ***P < 0.001

In HCC clinic, patients are limited by choice for effective treatment options. In order to identify functionally important TSGs and elucidate their signaling cascades in the hope of finding drug targets for HCC patients, we examined the DNA copy number variation (CNV) and focused our attention on deleted regions at whole genome level. Strikingly, we noticed a sharp peak of focal deletion in 8P23.1 chromosomal region across most HCC samples (Figure 1A, left). Further analysis revealed that the deletion centered around GATA4 gene locus (Figure 1A, right), a strong hint for GATA4 as a TSG. Consistently, TCGA data showed significantly lower mRNA expression of GATA4 in HCC tumor nodules in comparison to para-tumoral tissues (Figure 1B). Moreover, higher expression level of GATA4 in HCC tumors was significantly correlated with patients' longer survival of male patients of all stage (Figure 1C). Of note, the same trend was likewise highly significant in stage I HCC patients (HR=0.35, p=0.0099, Figure S1A). These data strongly suggested that GATA4 was a clinically relevant TSG.

In order to validate its TSG function, we sought to ectopically express GATA4 in HCC cell lines with lower baseline expression and knockdown in those with relatively higher expression. For this purpose, we checked GATA4 expression level in liver cancer cell lines commonly used in cancer research community through western analysis, including two pairs of HCC/para-tumoral tissues as references for GATA4 expression in tumoral and normal liver tissues. We found comparable GATA4 protein level between tumor samples and cancer cell lines, which was consistently lower than that of para-tumoral tissues (Figure S1B). Among the tumor cell lines, GATA4 protein level was relatively higher in SNU-387, SK-HEP1, NeHepLxHT and HUH-7 than in SNU-449, PLC-PRF-5 and HepG2 (Figure S1B). We generated HepG2 and Huh7 cell lines for doxycycline (DOX) inducible expression of GATA4 (designated HepG2i and Huh7i respectively) (Figure S1C) and found that ectopic expression of GATA4 significantly slowed down growth rate and inhibited the ability to form colonies in 2D-plate of HepG2i and Huh7i (Figure 1D and Figure S1D). Conversely, GATA4 knockdown significantly promoted growth rate and enhanced colony-forming ability of Huh7 and SNU387 (Figure 1E and Figure S1E-G). We also found that DOX treatment inhibited sphere-forming ability of HepG2i and Huh7i (Figure S1H & S1I). Taken together, these in vitro data strongly argued that GATA4 was a potent TSG for liver cancer.

To evaluate its TSG function in vivo, we sought to check the tumor formation after knockout of GATA4 in hepatocytes of transgenic mouse model. Previously, Jacks and colleagues reported a virial CRISPR/CAS9 system to simultaneously knockout a target gene and activate mutant Kras somatically in lsl-Kras^G12D^ transgenic mice 26 (Figure S1J). Using this system, we successfully activated Kras^G12D^ and knockout GATA4 simultaneously in hepatocytes of lsl-Kras^G12D^ transgenic mice (Figure S1K). Six months after virus infection, 3 out of 6 mice treated with pSECC-sgGATA4 lentivirus developed invasive liver tumors; In stark contrast, none of 6 pSECC-sgTdTomato lentivirus (serving as negative control) treated mice developed liver cancer (Figure 1F-H & Figure S1L).

Taken together, our data solidly showed that GATA4 was a functional and clinically relevant TSG for HCC.

### Ectopic expression of GATA4 resulted in cellular senescence of HCC cell lines

**Figure 2:**
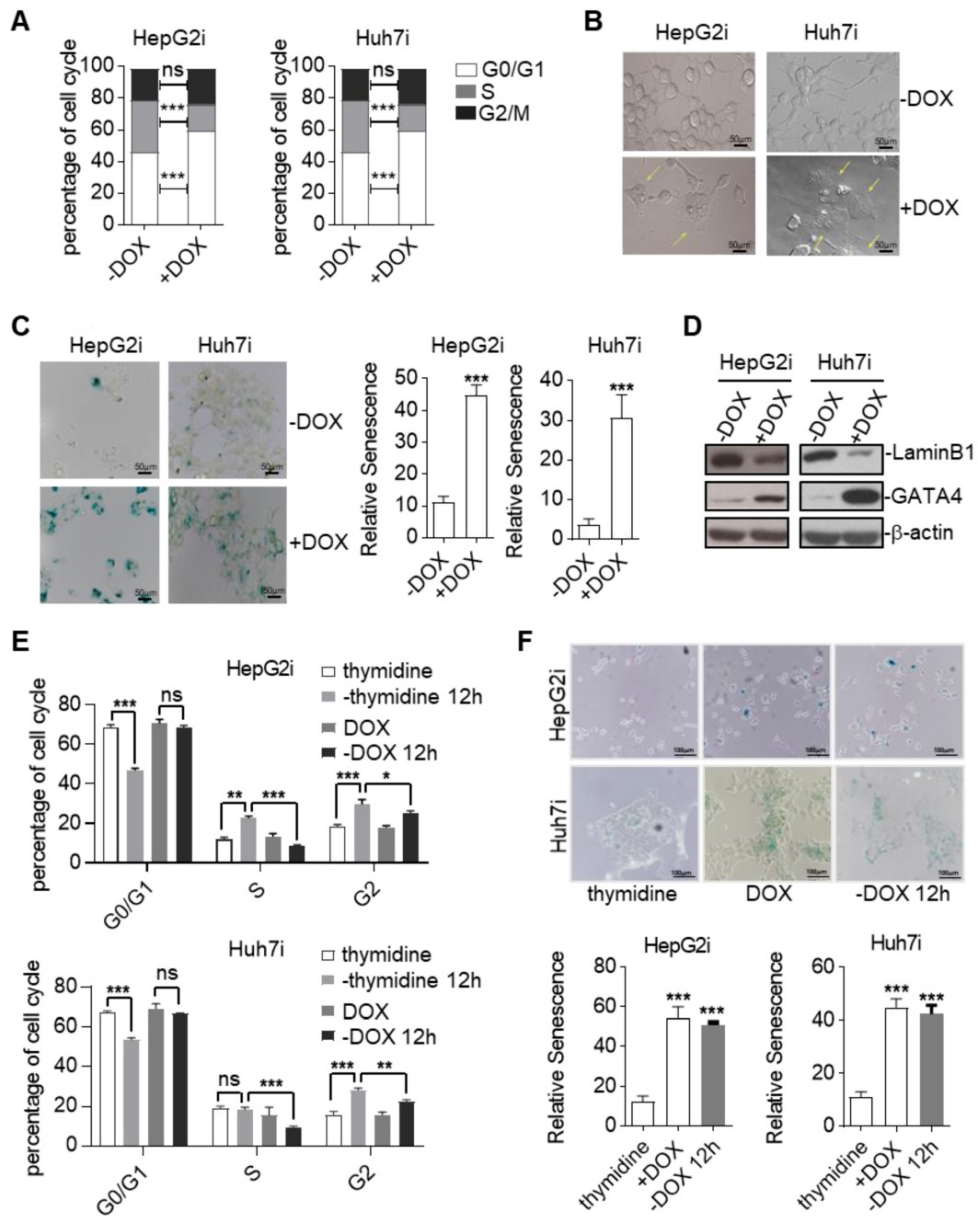
Ectopic expression of GATA4 resulted in cellular senescence of HCC cell lines. (a) GATA4 induced cell cycle arrest at G0/G1 in liver cancer cell lines. Representative cell cycle distribution was shown for HepG2i (left) and Huh7i (right) treated with or without DOX (1 mg/mL, 48 hours). (b) Morphology of liver cancer cells expressing GATA4. HepG2i cells and Huh7i cells were treated with or without DOX (1 mg/mL) for 48 hours (scale bars, 50 mm). (c) Senescence-associated β-galactosidase staining. HepG2i cells and Huh7i cells were treated with or without DOX (1 mg/mL) for 48 hours. **Left:** representative staining, **Right:** statistics of the positive percentage of senescence cells, (scale bars, 100 mm). (d) GATA4 reduced nuclear LaminB1 expression in liver cancer cell lines. HepG2i cells and Huh7i cells (2×10^6^) were left untreated or treated with DOX for 48 hours. Lysates of the nuclear extracts (LaminB1) or whole cell lysates (GATA4 and b-actin) were analyzed by immunoblots with the indicated antibodies. (e) GATA4 expression led to stable arrest of liver cancer cells at G0/G1 phase. Logarithmic phase cells (2×10^6^) were treated with 2 mM of thymidine or 1 mg/mL of DOX for 48 hours, followed by culturing in drug-free media for another 12 hours. Cell cycle distribution was monitored through FACS analysis. (f) Senescence-associated β-galactosidase staining of cells in **E**. **Upper:** representative images. **Lower:** Statistics of the positive percentage of senescence cells (scale bars, 100 mm). Data are representative of three independent experiments, and were analyzed by unpaired t-test. Error bars denote SD. *P < 0.05; **P < 0.01; ***P < 0.001

TSGs inhibit tumor formation mainly by inducing cell-cycle arrest, apoptosis, and senescence 27. In order to find out by which means GATA4 exerted its tumor suppressive function, we first checked cell death of HepG2 and Huh7 in response to GATA4 expression. FACS analysis of Annexin V and propidium iodide (PI) stained cells revealed no significant change of death rate before and after GATA4 expression in both cell lines (Figure S2A). Consistently, western analysis revealed no obvious cleavage of Caspase-3 and Caspase-9 before or after expression of GATA4 (Figure S2B). These results suggested that apoptosis was not involved during the process when GATA4 exerted its TSG function in these cell lines. Next, we checked the impact of GATA4 expression on cell cycle distribution. Interestingly, we found that GATA4 expression led to a significant increase of the percentage of G0/G1 cells and a concomitant decrease in S phase in both HepG2i and Huh7i (Figure 2A and Figure S2C). We also noticed that HepG2i and Huh7i cultured in DOX-containing media exhibited an enlarged, flattened and irregular shape (Figure 2B), a morphology typical of a senescent cell. We further confirmed GATA4-induced senescence of HepG2i and Huh7i through β-galactosidase staining (Figure 2C). In line with this, western analysis revealed that GATA4 expression led to downregulation of Lamin B1 in nuclei, a senescence associated biomarker^28^, in HepG2i and Huh7i (Figure 2D).

Senescence demarcates a cell of a stable exit of cell cycle such that a senescent cell doesn't reenter cell cycle even when stress has been cleared. As a functional validation of GATA4 induced senescence, we treated HepG2i and Huh7i cells with DOX for 48 hours to induce senescence, followed by culturing in DOX-free media for another 12 hours. FACS analysis showed that once treated with DOX, both cells were stably arrested in G0/G1 phase despite of prolonged culture in DOX-free media (Figure 2E, Figure S2D & S2E). This was in stark contrast to efficient cell cycle entry from G0/G1 observed in thymidine-induced cycle-arrested HepG2 and Huh7 when switched to thymidine-free media (Figure 2E, Figure S2D & S2E). Consistently, β-galactosidase staining confirmed positive senescent signals in HepG2i and Huh7i cell by the end of experiment even after washing out DOX for 12 hours (Figure 2F).

Cellular senescence is commonly induced by cyclin-dependent kinase (CDK) inhibitors (CKI). Our western blot analysis showed no significant changes of p14ARF, p15INK4b, p16INK4a, p21, p27 or p53 levels before and after GATA4 expression in HepG2i cells (Figure S2F).

Taken together, our data showed that GATA4 exerted its TSG function by inducing senescence of HCC cells through a non-canonical pathway.

### GATA4 interacted and colocalized with β-catenin

**Figure 3:**
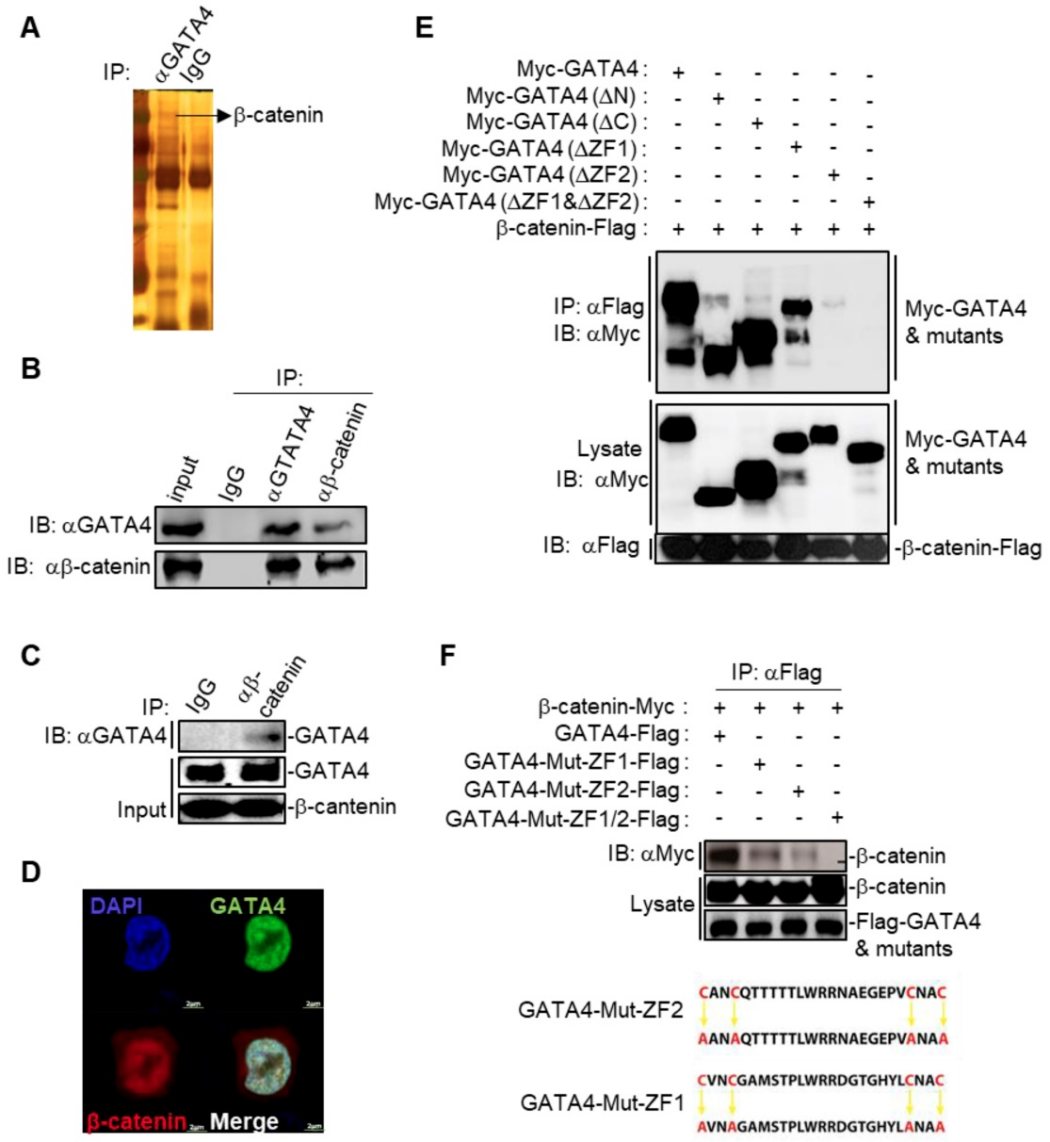
GATA4 interacted and colocalized with b-catenin. (a) Silver-staining of SDS-PAGE separated protein samples immunoprecipitated with antibody targeting with GATA4 or control IgG. (b) Endogenous GATA4 interacted with b-catenin in HepG2 cells. Coimmunoprecipitation experiments were performed with anti-GATA4 or anti-b-catenin, and the immunoprecipitates and whole cell lysate (input) were analyzed by immunoblots with indicated antibodies. (c) GATA4 directly bound to b-catenin. In vitro GST pull-down assay indicating a direct interaction between GATA4 and b-catenin. (d) Colocalization of GATA4 and b-catenin. HepG2 cells (1×10^6^) were co-transfected with constructs encoding GATA-GFP and b-catenin-mCherry respectively and analyzed with confocal microscopy 24 hours later (scale bars, 2 mm ). (e & f) Domain mapping of GATA4-b-catenin association. The HEK293 cells (2×10^6^) were transfected with the indicated plasmids (5 mg each). Coimmunoprecipitation and immunoblot were performed with the indicated antibodies. Mutations of ZF domains of human GATA4 were detailed in diagram (lower panel, Figure 3F).

We then asked the molecular mechanisms underlying the GATA4-induced senescence in HCC cell lines. We first checked the binding partners of GATA4 through immunoprecipitation (IP) in DOX treated HepG2i cells. Silver staining of the SDS-PAGE gel clearly revealed distinct protein species in GATA4 pull-down samples compared to IgG-enriched control (Figure 3A). We then explored putative interacting partners of GATA4 by LC-MS/MS and identified more than 100 protein species (Table S1). Earlier, Adams and colleagues reported that inhibition of Wnt signaling resulted in cell senescence 29. Interestingly, we detected several peptides of β-catenin with high confidence in GATA4 immunoprecipitant, a strong indication for interaction between GATA4 and β-catenin in HCC cells (Table S1). When co-overexpressed GATA4 and β-catenin in HEK293 cells, GATA4 efficiently pulldown β-catenin (Figure S3B). Likewise, ectopically overexpressed GATA4 readily precipitated endogenous β-catenin in HepG2 cells (Figure S3C). To further confirm the interaction between endogenous GATA4 and β-catenin in HCC cells, we harvested protein samples from 5×10^7^ HepG2 cells to enrich endogenous protein with antibodies against GATA4 and β-catenin respectively. Reciprocal co-IP experiments solidly confirmed binding between endogenous GATA4 and β-catenin in HepG2 cells (Figure 3B). Furthermore, we were able to confirm direct binding between GATA4 and β-catenin through GST-pulldown experiment using bacterially expressed product of GATA4 and β-catenin (Figure 3C and Figure S3A).

GATA4 is a transcription factor, which constitutively locates in nucleus 30. We went on to check whether GATA4 interacted with β-catenin in nuclei of liver cancer cells. We overexpressed EGFP fused GATA4 and mCherry fused β-catenin in HepG2 cells. Confocal microscopy revealed minor portion of mCherry-β-catenin fusion protein in cytoplasm, with majority in nucleus. In contrast, GATA4 exclusively located in nucleus. We found that GATA4 colocalized with β-catenin only in nucleus of HepG2 cells (Figure 3C).

We further asked which domain of GATA4 mediated the interaction between GATA4 and β-catenin. For this purpose, we ectopically expressed in HEK293 cells FLAG tagged β-catenin together with Myc tagged full-length GATA4 and its truncated mutants deleted of N-terminal TAD domains, CTR region, ZF1, ZF2, or ZF1/ZF2 respectively (Figure S3D). Our co-IP data solidly showed that while deletion of ZF1 slightly affected, deletion of ZF2 drastically diminished, the ability of GATA4 to bind β-catenin. Simultaneous deletion of ZF1 and ZF2 completely abolished the interaction between GATA4 and β-catenin (Figure 3E). To further confirm the above result and elucidate contribution of ZF1 and ZF2 to interaction between GATA4 and β-catenin, we expressed FLAG tagged ZF1 and ZF2 mutants of GATA4 and Myc tagged β-catenin in HEK293 cell and analyzed interaction. ZF domain features 4 conserved cystine residues 31 (Figure 3F). To avoid the possible impact of deletion of ZF domain on overall protein structure, which might negatively affect interaction between GATA4 and β-catenin, we expressed ZF mutants with all 4 conserved cystine residues replaced with alanine (C4A mutant) to minimize the impact on overall protein structure of GATA4 and checked the interaction between these mutants and β-catenin (Figure 3F). Consistently, we found that while C4A mutation of ZF1 slightly diminished, that of ZF2 severely inhibited this interaction (Figure 3F). Double C4A mutation of ZF1 and ZF2 completely lost its ability to interact with β-catenin (Figure 3F). Our data, therefore, solidly argued that ZF2 of GATA4 played a major role in mediating binding between GATA4 and β-catenin.

We also tried to pin down the domain of β-catenin critical for interaction between β-catenin and GATA4. We therefore generated a series of β-catenin mutant, lacking N-terminal domain (NTD), armadillo repeats (ARM), and C-terminal domain (CTD) respectively (Figure S3E). However, we were not successful in expressing ARM-deleted mutant of β-catenin (Figure S3F). Therefore, we were not able to reach a conclusion which domain in β-catenin was critical for interaction between β-catenin and GATA4.

### GATA4 inhibited canonical Wnt signaling by blocking β-catenin's recruitment of essential co-transcription factors

**Figure 4:**
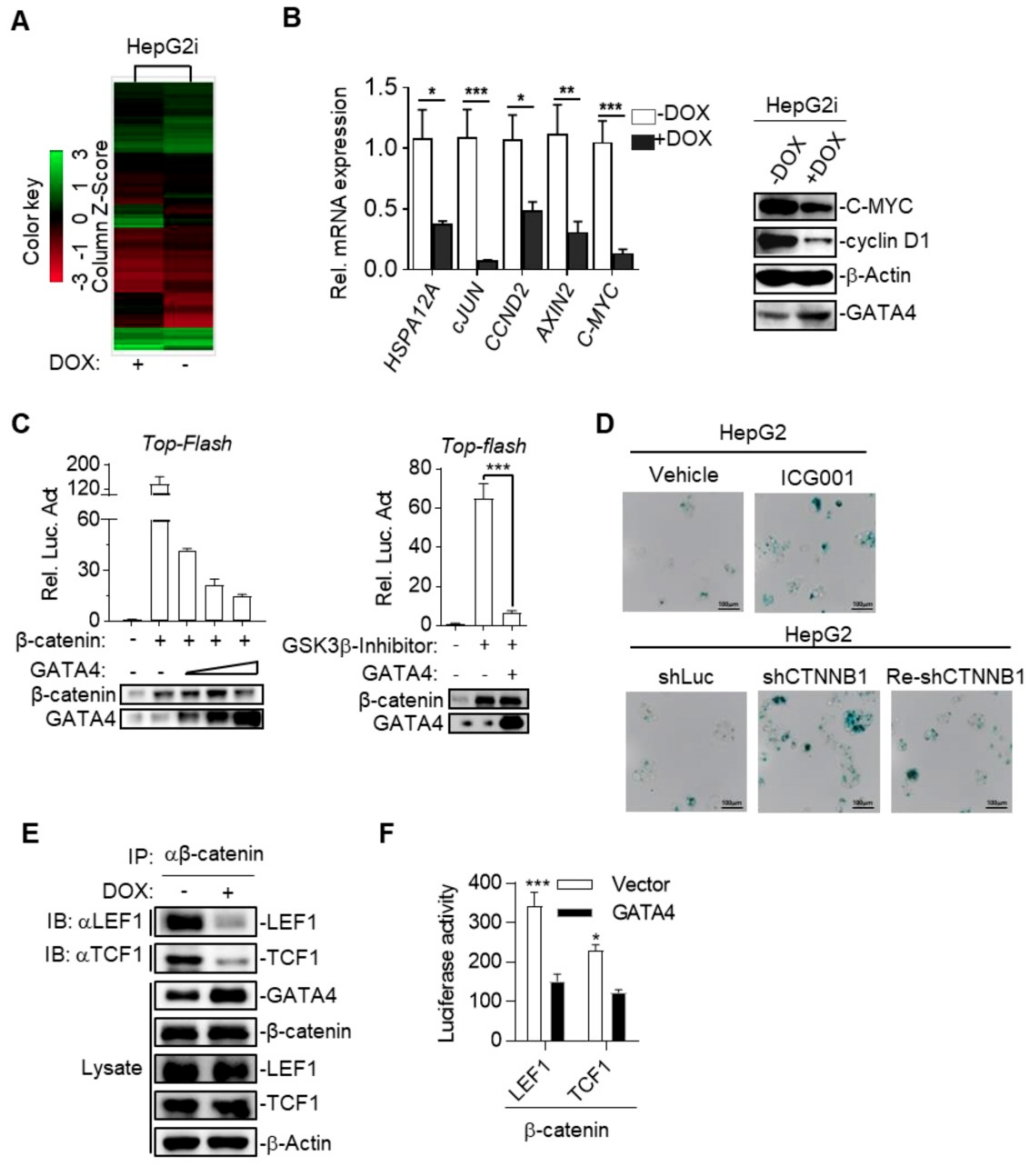
GATA4 inhibited canonical Wnt signaling by blocking β-catenin's recruitment of essential co-transcription factors. (a) Heatmap representation of the mRNA expression level between HepG2i cells treated with or without DOX. (b) GATA4 expression inhibited canonical Wnt signaling pathway. RNA was extracted from HepG2i treated w/o DOX (1 mg/mL) for 48 hours. Expression of the indicated genes was quantified through qPCR (**left**) or immunoblot analysis (**right**). (c) GATA4 inhibited transcriptional activity of b-catenin. **Left:** HEK 293 cells (1×10^5^) were transfected with the *Top-Flash* reporter plasmid (0.1 mg) and b-catenin expression plasmid(0.1 mg )together with increased amounts of GATA4 expression plasmid (0.05, 0.1, 0.2 mg), followed by monitoring luciferase 24 hours later. **Right:** HepG2 cells (1×10^5^) were transfected with the *Top-Flash* reporter plasmid and GATA4 expression plasmid. Cells were then left treated w/o GSK3b-Inhibitor for 12 hours before luciferase assays were performed. (d) **Upper:** Senescence-associated β-galactosidase staining of ICG001 and Vehicle treated HepG2 cells. HepG2 cells were treated w/o b-catenin inhibitor, ICG001 (0.1 mg/mL) for 48 hours. **Lower:** Senescence-associated β-galactosidase staining of HepG2 cells which harboring shLuciferase (shLuc), shCTNNB1 or HepG2 cells harboring shCTNNB1 transfected with PC3.1-CTNNB1 plasmid (Re-shCTNNB1), Scale bars:100mm. (e) GATA4 suppressed the interaction between b-catenin and LEF1/TCF1. HepG2i cells (5×10^7^) were treated w/o DOX for 48 hours followed by immunoprecipitation with anti-b-catenin antibody. The immunoprecipitants and whole cell lysate were analyzed by immunoblots with indicated antibodies. (f) Bimolecular fluorescence complementation assay determining the impact of GATA4 on interaction between b-catenin and LEF1 or TCF1. HEK 293 cells (1×10^5^) were transfected with the b-catenin-N-luciferase plasmid (b-catenin, 0.1 mg) and LEF1-C-luciferase (LEF1, 0.1 mg) or TCF1-C-luciferase (TCF1, 0.1 mg). Luciferase assays were performed 24 hours after transfection. Data are representative of three independent experiments, and were analyzed by unpaired t-test. Error bars denote SD. *P < 0.05; **P < 0.01; ***P < 0.001

To further study the mechanism underlying TSG function of GATA4 in HCC cells, we checked the alteration of gene expression at transcriptomic level through RNA-sequencing on HepG2i before or after DOX treatment. Our data showed that GATA4 expression upregulated 3903 genes and downregulated 2861 genes for more than 2 folds (Figure 4A and Table S2). GOTERM analysis showed that GATA4 expression resulted in significant changes of Wnt signaling pathway among various other pathways (Table S3). Quantitative reverse-transcription PCR (qRT-PCR) confirmed downregulation of canonical Wnt signaling pathway target genes, including *HSPA12A*, *cJUN*, *CCND2*, *AXIN2* and *C-MYC* (Figure 4B, left). Western analysis further confirmed downregulation of C-MYC and cyclin D1 at protein level (Figure 4B, right). Transcriptional activity of β-catenin is a critical readout of activity of canonical Wnt signaling 32. Top-flash assay confirmed that GATA4 expression inhibited transcriptional activity of β-catenin (Figure 4C). Of note, we observed similar trend of inhibitory function of GATA4 on mutant β-catenin frequently seen in liver cancer patients such as S45Y (Figure S4A). Importantly, knockdown and pharmacological inhibition of β-catenin both inhibited growth rate of HepG2 cells and resulted in senescence (Figure 4D and Figure S4B-G).

We then asked how GATA4 inhibited transcription activity of β-catenin. Typically, stabilized β-catenin accumulates in cytoplasm and translocates into nucleus to form complex with TCF1/LEF1 to drive expression of target genes^33^. Co-IP experiments revealed that DOX treatment of HepG2i cells efficiently suppressed the ability of endogenous β-catenin to pull down LEF1 or TCF1 (Figure 4E). This inhibition was further confirmed by our observation that ectopically overexpressed GATA4 also inhibited recruitment of LEF1 or TCF1 by β-catenin in HEK293 cells (Figure S4H & S4I). To quantitatively measure the ability of GATA4 to block interaction between β-catenin and LEF1/TCF1, we turned to bimolecular fluorescence complementation assay 34 by fusing β-catenin and LEF1/TCF1 to N- and C-terminal half of firefly luciferase respectively, such that when these fusion proteins were expressed in HepG2 cells, luciferase activity is a direct readout for interaction between β-catenin and LEF1/TCF1. Results showed that GATA4 potently inhibited interaction of β-catenin with LEF1/TCF1 (Figure 4F).

Taken together, our data showed that GATA4 bound to β-catenin, and thus prevented cofactors like LEF1/TCF1 from forming functional complex with β-catenin to transcribe target genes of canonical Wnt signaling pathway.

### GATA4 assembled a tumor suppressor enhancing module between itself and β-catenin

**Figure 5:**
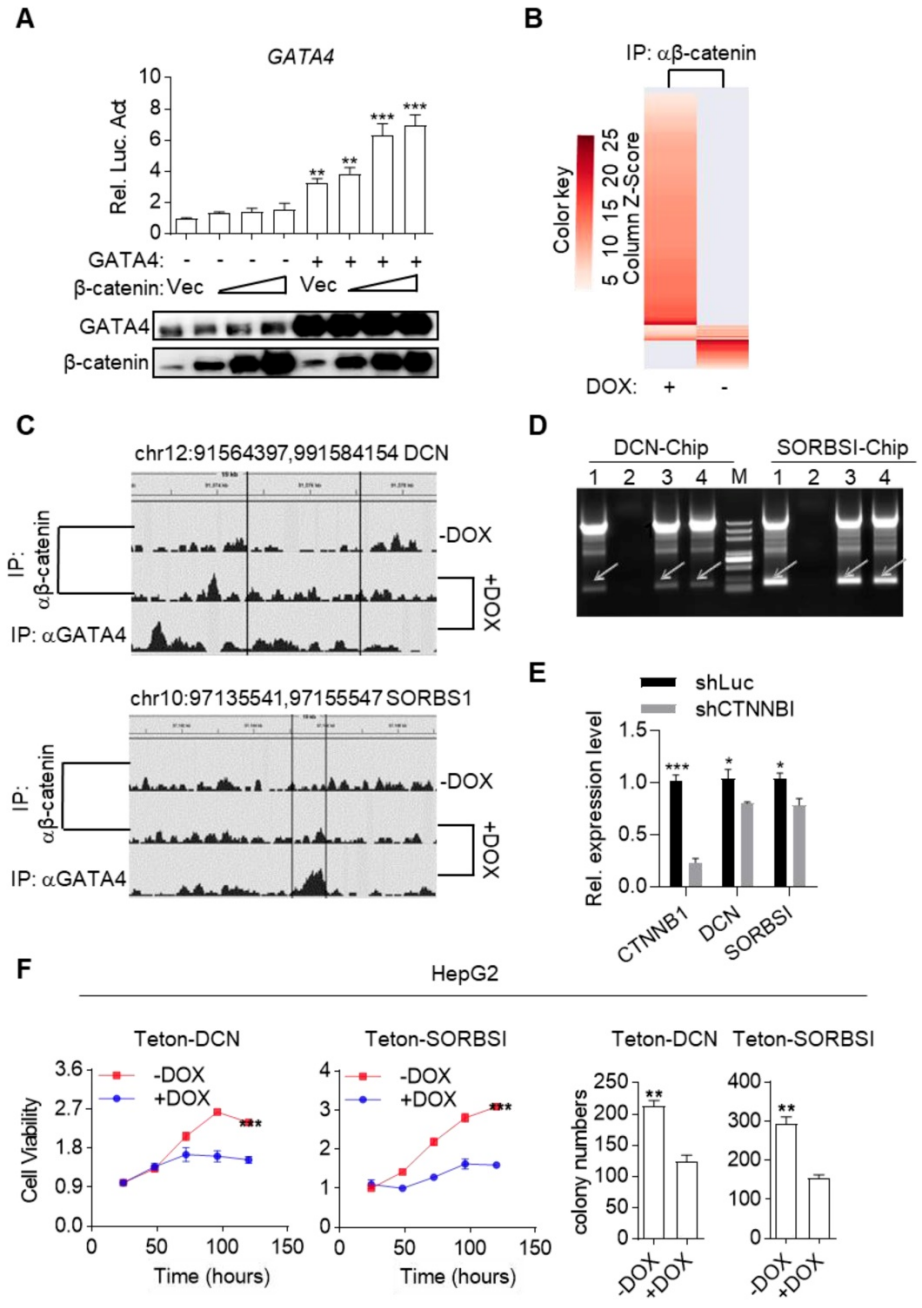
GATA4 assembled a tumor suppressor enhancing module between itself and β-catenin. (a) b-catenin enhances *GATA4* transcriptional activity in a dose-dependent manner. HEK 293 cells (1×10^5^) were transfected with the *GATA4* reporter plasmid (0.1 mg) and increased amounts of b-catenin expression plasmid (0.05, 0.1, 0.2 mg) together w/o GATA4 expression plasmid(0.2 mg). Luciferase was monitored 24 hours later. (b) ChIP-seq result of DNA fragments enriched with antibody targeting b-catenin from HepG2i cells cultured w/o DOX. (c) b-catenin was recruited to GATA4 chromosome binding site. HepG2 cells (1.6×10^8^) were treated w/o DOX (1 mg/mL) treatment. ChIP-Seq was conducted on DNA samples enriched with indicated antibodies from indicated cells. (d) b-catenin bound promoter region of DCN and SORSBI genes in the presence of GATA4. 1: input, 2: b- catenin enriched DNA elements in HepG2i cells in absence of DOX, 3: b-catenin enriched DNA elements in HepG2i cells in the presence of DOX, 4: GATA4 enriched DNA elements in HepG2i cells in the presence of DOX. (e) β-catenin promoted GATA4 to transcribe DCN and SORBS1 expression. *CTNNB1*, *DCN* and* SORBS1* mRNA level in DOX treated HepG2i cells after transfected with shRNA-luc or shRNA-CTNNB1. (f) DCN and SORSBI inhibited cell proliferation. HepG2-Teton-DCN and HepG2-Teton-SORBS1 cells were treated w/o DOX (1 mg/mL) for indicated time points followed by monitoring viability with CCK-8 reagents (**Left**) and statistics of colony formation (**Right**). Data are representative of three independent experiments, and were analyzed by unpaired t-test. Error bars denote SD. *P < 0.05; **P < 0.01; ***P < 0.001

Surprisingly, we inadvertently found that β-catenin enhanced transcriptional activity of GATA4 during our study. As shown in Figure 5A, β-catenin dose-dependently enhanced GATA4 reporter activity in HEK293 cells in the presence of GATA4 expression. This data, together with our data in Figure 3 and Figure 4, suggested that binding of GATA4 to β-catenin created an interesting tumor suppressor enhancing module: 1) GATA4 prohibited β-catenin from transcribing canonical Wnt target genes (like *Cyclin D1* and *C-MYC* et.al.) and thereby inhibited the oncogenic function of β-catenin; 2) β-catenin enhanced transcription activity of GATA4 to exert tumor suppressor function. Given the fact that several high-efficiency systems for delivering genes-of-interest into target histocytes and tumor cells were developed or in clinical trials^35^, ^36^, this tumor suppressor enhancing module is of high significance as it could be therapeutically exploited in clinic by ectopically expressing GATA4 in HCC tissues. To further study the molecular mechanism of this tumor suppressive module, we performed β-catenin ChIP-seq to profile genome-wide β-catenin binding patterns in DOX treated or untreated HepG2i cells (Table S4 and S5). We found that GATA4 expression drastically changed the DNA-binding pattern of β-catenin in HepG2i cells (Figure 5B). As exemplified in Figure 5C, DOX treatment resulted in relocation of β-catenin from its original binding sites to GATA4 binding sites. Interestingly, we found GATA4 binding consensus sequences in both DNA sequences immunoprecipitated with β-catenin antibody shown in Figure 5C, but no β-catenin binding site in either of them (Figure S5B).

We went on to validate whether β-catenin enhanced GATA4's transcription of TSG targets. We re-analyzed our RNA-seq data and found several liver cancer TSGs among GATA4-upregulated genes (Table S6). We picked DCN and SORBS1 for further validation. ChIP-PCR confirmed that β-catenin by itself didn't bind their promoter sequences while GATA4 did. However, β-catenin bound their promoter sequence in the presence of GATA4 (Figure 5D). Moreover, qRT-PCR showed that knockdown of β-catenin diminished expression level of DCN and SORBS1 in DOX treated HepG2i cells (Figure 5E). Ectopic expression of DCN and SORBS1 inhibited HepG2 cell growth (Figure 5F and Figure S5A) and colony formation in 2-D plates (Figure 5F and Figure S5C) respectively, validating their tumor suppressor function. Thus, we validated a tumor suppressor enhancing module assembled by ectopically expressed GATA4 in HCC cells. This module functions to not only prevent β-catenin from transcribing canonical Wnt signaling target genes to support liver cancer cell growth, but also recruit β-catenin to facilitate transcription of TSGs by GATA4 in HCC cells. Reflecting the involvement of this module in liver cancer patients, we observed that frequency of CTNNB1 mutation tend to be higher in GATA4-low patients than in GATA4-high cohorts (Figure S5D).

### GATA4 deficiency denotes an opportunity for therapeutic intervention of HCC

**Figure 6:**
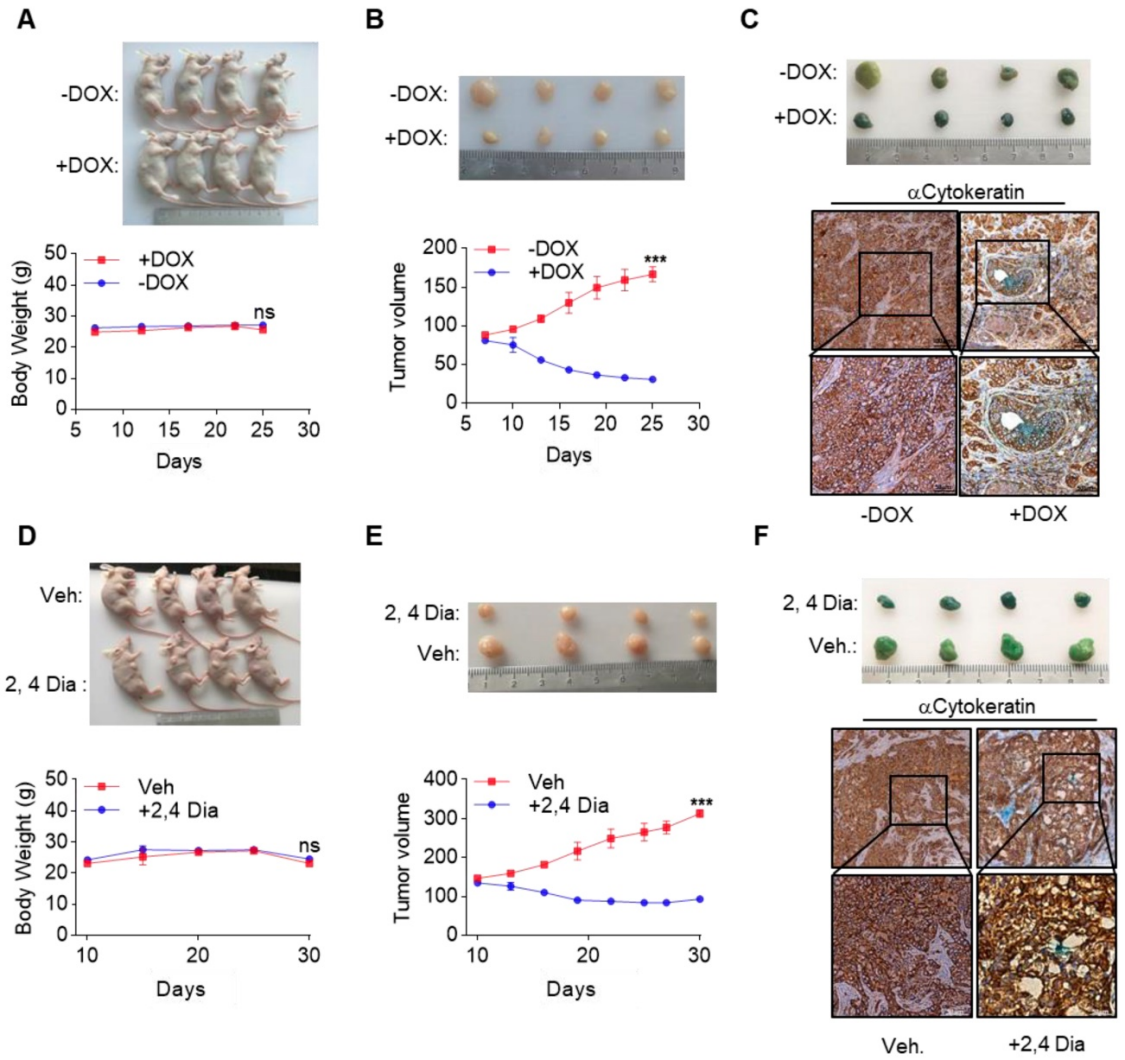
GATA4 deficiency denotes an opportunity for therapeutic intervention of HCC. (a & b) GATA4 expression shrank HepG2 xenografted tumors. Four mice in each group were treated according to schedules outlined in (Figure S6A). Picture of the mice were shown in upper panel. Body weight of the mice was recorded every 5 days and graphed in lower panel. Tumor growth was recorded every 2 days by measuring its diameter with Vernier caliper in the mice detailed in (a). Tumor volume was calculated by tumor volume (mm^3^) = D×d^2^/2, where D is the longest and d is the shortest diameter respectively (b). (c) GATA4 promoted cell senescence in tumor. b-galactosidase staining was conducted on tumors collected from the mice treated w/o DOX food. The stained tumor nodules were subjected to IHC staining with Cytokeratin (AE1/AE3) antibody (scale bars:100mm). (d & e) 2,4 Dia treatment shrank HepG2 xenografted tumors. Four mice in each group were treated according to schedules outlined in **(**Figure S6B**)**, picture of the mice shown in upper panel. Body weight of the mice was recorded every 5 days and graphed in lower panel (d). Tumor growth was recorded every 3 days (e). (f) 2,4 Dia treatment resulted in senescence of cells of HepG2 xenografted tumors. b-galactosidase staining was conducted on tumors collected from the mice treated with 2,4 Dia or Vehicle. The stained tumor nodules were subjected to IHC staining with Cytokeratin (AE1/AE3) antibody (scale bars:100mm). Data are representative of three independent experiments, and were analyzed by unpaired t-test. Error bars denote SD. *P < 0.05; **P < 0.01; ***P < 0.001

Currently, very few effective treatment options are available for late-stage HCC patients. Our data suggested that by orchestrating a tumor suppressor enhancing module, ectopic expression of GATA4 in HCC cells might be a valid choice to shrink HCC tumors. We then went on to test this hypothesis in preclinical settings. We inoculated HepG2i cells subcutaneously in nude mice and randomized them for treatment with DOX-containing or control diet when tumors reached a volume of 90 mm^3^ (Figure S6A). Interestingly, while tumors in control-diet-fed mice continued growing, significant tumor shrinkage was seen in DOX-treated group (Figure 6A, upper). Of note, DOX treatment was not toxic as indicated by constant weight of mice during the experiment (Figure 6A, lower). On day 26 post-treatment, the average volume of tumor in DOX treated group is around 30 mm^3^, in stark contrast to an average of around 160 mm^3^ in the control diet group (Figure 6B). β-galactosidase staining revealed robust senescence of tumor cells in DOX treated group and largely negative in control group (Figure 6C). Given the facts that GATA4 is almost ubiquitously deficient in HCC patients and that several highly efficient systems for delivering genes for overexpression in tumor nodules are under development or clinical trial^35^, ^36^, our current finding is of paramount translational importance.

Meanwhile, β-catenin is frequently hyperactive in liver cancers. We reasoned that deficiency of GATA4 in liver cancer cells unleashed the oncogenic activity of β-catenin. In this scenario, inhibition of β-catenin might partially mimic biological effect of GATA4 expression in HCC cells. Consistent with this hypothesis, we found a significant reverse correlation between expression level of GATA4 and some WNT/β-catenin target genes (Figure S6D). We then went on to treat HCC tumors with β-catenin inhibitors. We inoculated HepG2 cells in nude mice and started treatment with 2,4-Dia, a potent inhibitor of β-catenin/Tcf-4^37^, when tumors reached around 120 mm^3^ (Figure S6B). We confirmed that just like ICG001, 2,4-Dia was able to induce HepG2 cell senescence (Figure S6E). Treatment with 2,4-Dia significantly shrank tumor (Figure 6D, upper and Figure 6E). Of note, our dosing scheme didn't result in obvious toxicity during treatment for 20 days (Figure 6D, lower). β-galactosidase staining revealed that 2,4-Dia treatment resulted in senescence of tumor cells, which was not seen in vehicle treated tumors (Figure 6F).

Taken together, our results suggested that deficiency of GATA4 may offer therapeutic opportunities for a large number of HCC patients.

## Discussion

HCC is the major pathological type of liver cancer, one of the most devastating tumors world-wide. Very few treatment options are available to late-stage HCC patient. Here we reported a previously unnoticed tumor suppressor enhancing module which could potentially be exploited in clinic for treating GATA4 deficient HCC patients. Given the fact that GATA4 is deficient in majority of HCC patients, our work shed light on a new therapeutic opportunity for these large number of patients.

In our current work, we found GATA4 chromosome segment was almost ubiquitously deleted in all HCC patients. We have functionally validated tumor suppressor function of GATA4. Indeed, a recent study showed that GATA4 was a tumor suppressor gene for HCC 38. This implies that finding a targeting therapy for GATA4 deficient HCC patients is of paramount clinical significance. Of note, tumor suppressive function of GATA4 was recently reported in other cancers 39.

β-catenin has been well known as an oncogene through transcribing target genes of canonical Wnt signaling pathway. Tumor promoting function of β-catenin has been repeatedly validated: oncogenic mutations in CTNNB1 frequently found in HCC patients; therapeutic effect in preclinical tumor models has been reported with drug targeting β-catenin. In our current study, we discovered an unexpected role for β-catenin in HCC: in the presence of GATA4 expression, β-catenin was recruited by GATA4 to facilitate transcription of GATA4 target genes, among which are HCC tumor suppressor genes. Our current work therefore unveiled a previously unnoticed tumor suppressor enhancing module orchestrated by ectopically expressed GATA4 in HCC cells: 1). GATA4 inhibited transcriptional activity of β-catenin, thereof the expression of canonical Wnt target genes (like *cyclin D1* and *C-MYC* et.al.), which inhibited the oncogenic function of β-catenin; 2). β-catenin was recruited by GATA4 and thereby enhanced transcription activity of GATA4 to exert tumor suppressor function. Given the promising virial and non-virial systems to deliver gene-of-interest into target histocytes and tumor cells^35^, ^36^, ^40^, over expressing GATA4 in HCC tumor cells is expected to elicit dramatic shrinkage of HCC tumors through two ways: suppression of the function of β-catenin; expression of tumor suppressor genes. Both effects bear hope to shrink HCC tumors and benefit patients. Our study also implied that GATA4-deficient HCCs may have higher canonic Wnt signaling activity. Our preclinical study has shown the safety and efficacy of 2,4-Dia, a drug currently in clinical trial, for this type of HCC. Meanwhile, our work also presents an interesting case that an oncogene conditionally functions as a TSG in the presence of another TSG.

Our current study suggests that further effort is worthwhile to study the status of GATA4 as a biomarker for treating HCC patients. Given the high frequency of GATA4 deficiency among HCC patients, our current work is of paramount translational significance.

## Materials and Methods

### Ethnics statement

All mice were housed in a pathogen-free environment at the Jinan University. All experimental protocols were approved by the Institutional Committee for Animal Care and Use at Jinan University. All animal work was performed in accordance with the approved protocol.

The protocol for collecting tumor samples was approved by Sun Yat-sen University Cancer Center in Guangdong, China. Written consent was obtained from every patient who donated tumor samples. All work was performed in accordance with the approved protocol.

### 2.1 Constructs

Following shRNAs were purchased from the Sigma Mission shRNA Library: shGATA4 (TRCN0000020424), shCTNNB1 (TRCN0000314920), and Luciferase shRNA (shLuc, SHC007). psPAX2, pMD2.G, pCAG-IRES-Neo and pLKO.1-puro were purchased from Addgene. The pLVX-TetOne-Puro plasmid was purchased from Clontech. The Top-Flash plasmid was a gift from Dr. Wei Wu's lab at Tsinghua University. Human SORBS1, DCN and CTNNB1 cDNA were kindly gifted by Dr. J. Han's lab at Xiamen University. Myc- or FLAG-tagged GATA4, β-catenin, TCF1, LEF1, all truncations and point mutations were constructed by standard molecular biology techniques.

### 2.2 Reagents and antibodies

Doxycycline hyclate (DOX, Sigma); ICG-001 (Selleckchem); fetal bovine serum, DMEM and DMEM/F12 medium (Gibco); fibroblast growth factor (FGF) and epidermal growth factor (EGF) (PeproTech); Trizol reagent (TAKARA); protease and phosphatase inhibitor cocktail (Roche); Lipofectamine 3000 and B27 (Invitrogen); dual-specific luciferase assay kit (Promega); Cell Counting Kit-8 (CCK8, Dojindo Molecular Technologies); Western blotting substrate (Millipore); Cell Signaling Senescence β-Galactosidase Staining Kit (CST); silver staining Rapid silver staining kit's (Beyotime); BALB/c nude mice (Beijing Vital River Laboratory Animal Technology); antibody against GATA4, Lamin B1, P21, FLAG, P15 and c-MYC (Abcam); HA and β-actin (Sigma); β-catenin, LEF1, TCF1, P14, P27, P53, p14/ARF, Caspase-9 and Caspase-3 (CST), P16/Ink4a (Epitomics); Cytokeratin (AE1/AE3) antibody (Kit-0009, MXB Biotechnologies) were purchased from the indicated manufacturers. SNU-387, SNU-449, PLC, NeHepLxHT, SK-Hep1, HepG2, HUH7 and HEK293 cells were obtained from ATCC.

### 2.3 Generation of engineered cell lines

The HEK293 cells were transfected with two packaging plasmids (psPAX2 and pMD2.G) together with a pLVX-TetOne-Puro vector control or the same vector constructs for expressing GATA4, SORBS1, or DCN, or shGATA4, shLuciferase, shCTNNB1 respectively. Twenty-four hours later, cells were changed with new medium for another 24 hours. The recombinant virus-containing medium was filtered and used to infect cells in the presence of polybrene (8 μg/mL). The infected cells were selected with puromycin (0.5 μg/mL) for seven days before further experiments.

### 2.4 Cell proliferation assay

For cell proliferation assays, 1×10^3^ cells were seeded in each well of 96-well plates and cultured overnight. DMEM containing 10% FBS medium was supplemented with 1 µg/mL DOX or 0.1 µg/mL ICG-001 for 1, 3, and 5 days. Proliferation activity was then determined using CCK8 cell counting kit following the manufacturer's protocol.

### 2.5 Colony formation assay

For each cell line, 1×10^3^ cells were plated in triplicate into 6 well plate containing culture medium with 10% FBS. Cells were grown for 14 days, then fixed with 4% formaldehyde in 1x PBS and stained with crystal violet. Colonies with the diameter larger than 200 µm were counted.

### 2.6 Sphere formation assay

For each cell line, 1×10^3^ cells were seeded in Ultra-Low attachment 6 well plate in serum-free conditioned medium DMEM/F12 medium, 20 μl/mL B27 supplement, 20 ng/mL FGF and 20 ng/mL EGF. After 2~4 weeks, tumor spheres with the diameter over 100 µm were counted.

### 2.7 RNA extraction and real-time RT-PCR

Total RNA was extracted using Trizol reagent and subjected to real-time PCR analysis to measure mRNA levels of the indicated genes. Data shown are the relative abundance of the indicated mRNA normalized to that of *GAPDH*. Gene-specific primer sequences were as follows.

AXIN2-Forward: AAATAACCCCTCAGAGCGATG

AXIN2-Reverse: TTCCAGTTCCTCTCAGCAATC

CCND2-Forward: ACTTGTGATGCCCTGACTG

CCND2-Reverse: ACTTGGATCCGTCACGTTG

CTNNB1-Forward: CACAAGCAGAGTGCTGAAGGTG

CTNNB1-Reverse: GATTCCTGAGAGTCCAAAGACAG

DCN-Forward: GCTCTCCTACATCCGCATTGCT

DCN-Reverse: GTCCTTTCAGGCTAGCTGCATC

HSPA12A-Forward: GGCTGGAGGTAGAAGGTGGA

HSPA12A- Reverse: GCGTTATTCCTGTGTCCCCA

SORBS1-Forward: TATCAGCCTGGCAAGTCTTCCG

SORBS1-Reverse: CCCGTCTGATTCCCTCTTCACT

JUN-Forward: GCTGCTCTGGGAAGTGAGTT

JUN-Reverse: TTTCTCTAAGAGCGCACGCA

MYC-Forward: GGACCCGCTTCTCTGAAAG

MYC- Reverse: GTCGAGGTCATAGTTCCTGTTG

GAPDH-Forward: GAAGGTGAAGGTCGGAGTC

GAPDH-Reverse: GAAGATGGTGATGGGATTTC

### 2.8 Protein extraction and immunoblotting

Whole cell lysates were extracted by using the NP-40 lysis buffer (20 mM Tris·HCl, pH 7.4, 150 mM NaCl, 1 mM EDTA, 1% Nonidet P-40, protease and phosphatase inhibitor cocktail), protein concentrations were determined by the Bradford assay. Soluble proteins (30~40 μg) were subjected to SDS-polyacrylamide gel electrophoresis. Coimmunoprecipitation and immunoblot analysis were then performed.

### 2.9 In vivo xenograft model

6-weeks-old male BALB/c nude mice (Beijing Vital River Laboratory Animal Technology Co., Ltd.) were used for in vivo animal experiments. The animals were housed in constant laboratory conditions of a 12 hours light/dark cycle and specific pathogen-free conditions. For xenograft study, mice were inoculated subcutaneously into the right-back with 5×10^6^ HepG2i cells in 100 µL PBS and Matrigel (1:1). When tumors reached volume of around 90 mm^3^, the mice were randomly grouped and fed with either DOX containing or normal diet; or gavaged with 100 μL of 2,4-Dia suspended in 0.5% sodium carboxymethyl cellulose (Na-CMC, 100 mg/kg) or vehicle every other day. The body weight and tumor volume (= D×d^2^/2 (mm^3^), where D is the longest and d is the shortest diameter, respectively) were monitored as indicated in Figure 6 for 1-2 weeks up to the end of the experiment. At the end of treatment, mice were sacrificed and tumors were collected, photographed and weighed.

### 2.10 Senescence associated β-galactosidase staining assay

β-galactosidase staining was performed using the Cell Signaling Senescence β-Galactosidase Staining Kit. Briefly, 2×10^5^ cells were seeded in each well of 6 well plates and treated as indicated. The cells were cultured until the time of staining. For quantification of β-galactosidase staining positive cells, the blue positive cells in at least three randomly selected fields at 200×magnification under an inverted microscope were counted.

### 2.11 RNA-sequencing and ChIP-sequencing

The related material, method and raw data are described in GEO website of accession number: GSE135579 (RNA-seq) & GSE135714 (ChIP-seq).

### 2.12 Transgenic model mouse care and use

All mice were housed in a pathogen-free environment in Jinan University and all experimental protocols were approved by the Institutional Committee for Animal Care and Use at Jinan University. All animal work was performed in accordance with the approved protocol. We used recombinant lentivirus co-expressing Cre and CRISPR/CAS9 to infect transgenic mouse model: Lsl-KRAS^G12D^ through tail vein injection. Liver tumor formation in KRAS^G12D^/GATA4-/- mice compared to KRAS^G12D^/TdTomato-/- mice 6 months post-infection.

## Supplementary Material

Supplementary figures, tables, and methods.
