# Supplementary material for: A tumor suppressor enhancing module orchestrated by GATA4 denotes a therapeutic opportunity for GATA4 deficient HCC patients
Source: Theranostics. 2020 Jan 1;10(2):484–97. doi: 10.7150/thno.38060 (PMC6929984; doi:10.7150/thno.38060)
Supplement: Supplementary file 1 — Supplementary figures, tables, and methods. [file thnov10p0484s1.pdf]

# A tumor suppressor enhancing module orchestrated by GATA4 denotes a therapeutic opportunity for GATA4 deficient HCC patients

Feng Lu[1*], Qian Zhou[1*#], Lu Liu[1*], Guandi Zeng[1], Weimin Ci[2], Wanting Liu[1], Gong Zhang[1], Zhiyi Zhang[1], Peng Wang[3], Aiqun Zhang[4], Yunfei Gao[5#], Lei Yu[6#], Qingyu He[1#], Liang Chen[1,7#]

[1] Key Laboratory of Functional Protein Research of Guangdong Higher Education, Institute of Life and Health Engineering, College of Life Science and Technology, Jinan University, Guangzhou 510632, China.

[2] Beijing Institute of Genomics, Chinese Academy of Sciences, Beijing 100101, China

[3] Beijing Ditan Hospital, Beijing 100015, China

[4] The General Hospital of People's Liberation Army (301 Hospital), Beijing 100853, China

[5] The First Affiliated Hospital, Biomedical Translational Research Institute and School of Pharmacy, Jinan University, Guangzhou 510632, China

[6] Beijing Tongren Hospital, Capital Medical University, Beijing 100730, China

[7] Lead Contact

* These authors contributed equally to this work

**Keywords:** targeting therapy, tumor suppressor gene, GATA4, mouse model, signaling transduction

Lu et al., Figure S1

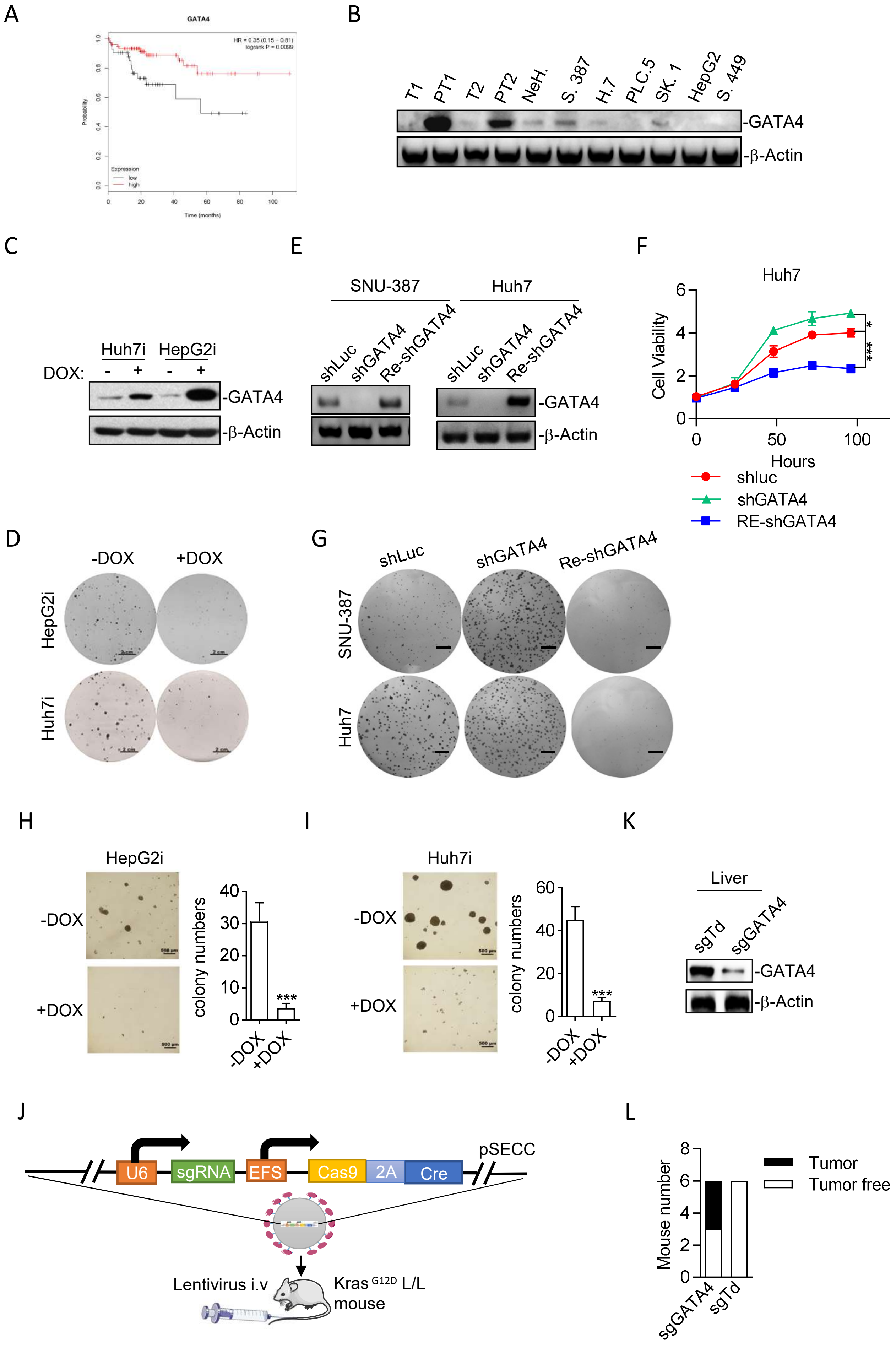

**Figure S1.** GATA4 is tumor suppressor gene in HCC.

**(A)** K-M survival analysis in patient (male, stage1, n=120) of liver cancer. **(B)** Immunoblot analysis of GATA4 expression in liver cancer cell lines along with clinical samples. T1: Tumor 1, PT1: paratumor1, T2: Tuomr2, PT2: paratumor2, NeH.: NeHepLxHT, S. 387: SNU-387, H.7: HUH-7, PLC.5: PLC-PRF-5, SK. 1: SK-HEP1, S. 449: SNU-449. **(C)** Effects of DOX induced expression of GATA4 in Huh7i and HepG2i stable cell line. Whole-cell lysates from the indicated cells were analyzed by immunoblots with the indicated antibodies. **(D)** Representative images of colony-forming assay of HepG2i and Huhi7 cells. **(E)** Effects of GATA4-shRNA plasmids on endogenous GATA4. Whole-cell lysates from the indicated cells were analyzed by immunoblots with the indicated antibodies. **(F)** Viability of Huh7 cells harboring shLuciferase (shLuc), shGTA4 transfected with PC3.1-GATA4 plasmid (Re-shGATA4) were assayed with CCK8 reagents for the indicated time points. **(G)** Representative images of colony-forming assay of SNU-387 and Huh7 cells. **(H& I)** GATA4 inhibited sphere formation ability of HepG2 and Huh7 cells. Representative images of sphere assay of HepG2i and Huh7i cells (left) and statistics of sphere formation(right). **(J)** Schematic representations of pSECC-Cre-Cas9-sgRNA. $Kras^{lsl\text{-}G12D/+}$ **(K)** GATA4 expression level in livers of mice at 6 months after lentivirus infection. (L) Numbers of tumor-bearing mice, $Kras^{lsl\text{-}G12D/+}/GATA4^{-/-}$ mice (sgGATA4) compared to $Kras^{lsl\text{-}G12D/+}/TdTomato^{-/-}$ mice (sgTd) 6 months post-infection. Six mice in each group.

Data are representative of three independent experiments, and were analyzed by unpaired t-test. Error bars denote SD. *P < 0.05; **P < 0.01; ***P < 0.001

Lu et al., Figure S2

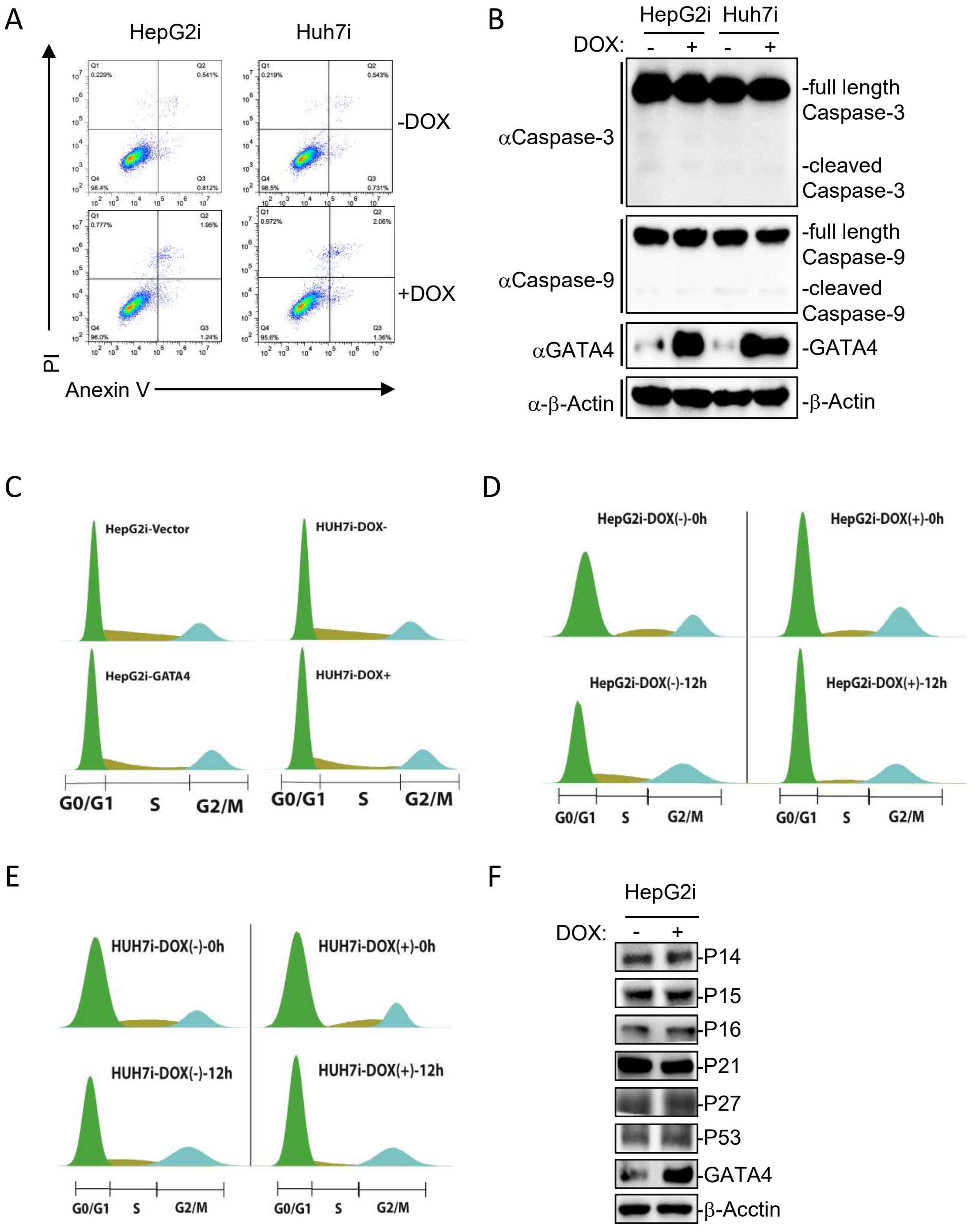

**Figure S2.** Effects of GATA4 on other signaling pathways.

**(A)** FACS analysis of annexin-V and propidium iodide (PI) staining of apoptotic cells following DOX treatment. The indicated cells ($1 \times 10^5$) were treated with DOX (1 μg/mL) for 48 hours before FACS analysis. **(B)** GATA4 was dispensable for apoptosis in liver cancer cells. HepG2i cells ($2 \times 10^6$) and Huh7i cells ($2 \times 10^6$) were left untreated or treated with DOX for 48 hours. Cell lysates were analyzed by immunoblots with the indicated antibodies. **(C, D & E)** Cell cycle distribution of HepG2i (left) and Huh7i (right) cells by FACS analysis. **(F)** Typical executor of cell senescence was not involved in GATA4-induced senescence. HepG2i cells ($2 \times 10^6$) were treated with or without DOX for 48 hours. Cell lysates were analyzed by immunoblots with the indicated antibodies.

# Lu et al., Figure S3

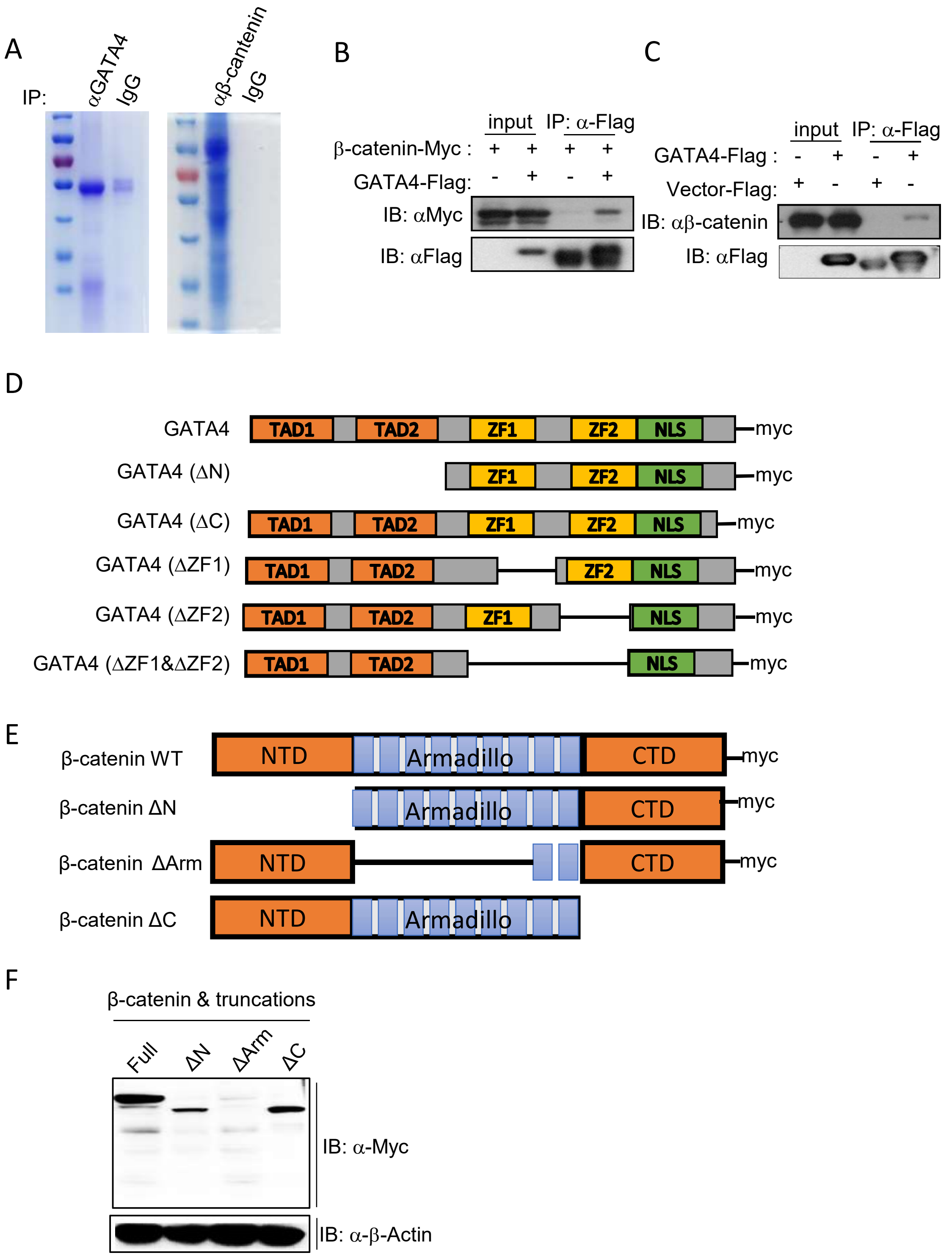

**Figure S3.** GATA4 interacted with β-catenin in liver cancer cells.

**(A)** Purified recombinant β-catenin or GATA4 proteins produced in E. coli are analyzed by SDS-PAGE and Coomassie staining. **(B)** Flag-GATA4 and myc-β-catenin were co-transfected into HEK293 cells ($2\times10^6$). After 30h, the cells were collected and lysed with RIPA Buffer. Then 10 μl flag-m2 beads were added to enrich the exogenously expressed flag-GATA4 protein. Finally, the interaction between β-catenin and GATA4 proteins was detected by western bloting. **(C)** Flag-vector and flag-GATA4 were exogenously expressed in HEK293 cells. Flag-m2 beads were used to enrich them. Then western blot was used to detect the interaction between endogenous β-catenin and exogenous GATA4 proteins**.** **(D)** A schematic representation of GATA4 and its truncated mutants. **(E)** A schematic representation of β-catenin and its truncation mutants. **(F)** Expression of truncated mutants of β-catenin. The HEK293 cells ($2\times10^6$) were transfected with the indicated plasmids (5 μg each). Immunoblot were performed with the indicated antibodies.

Lu et al., Figure S4

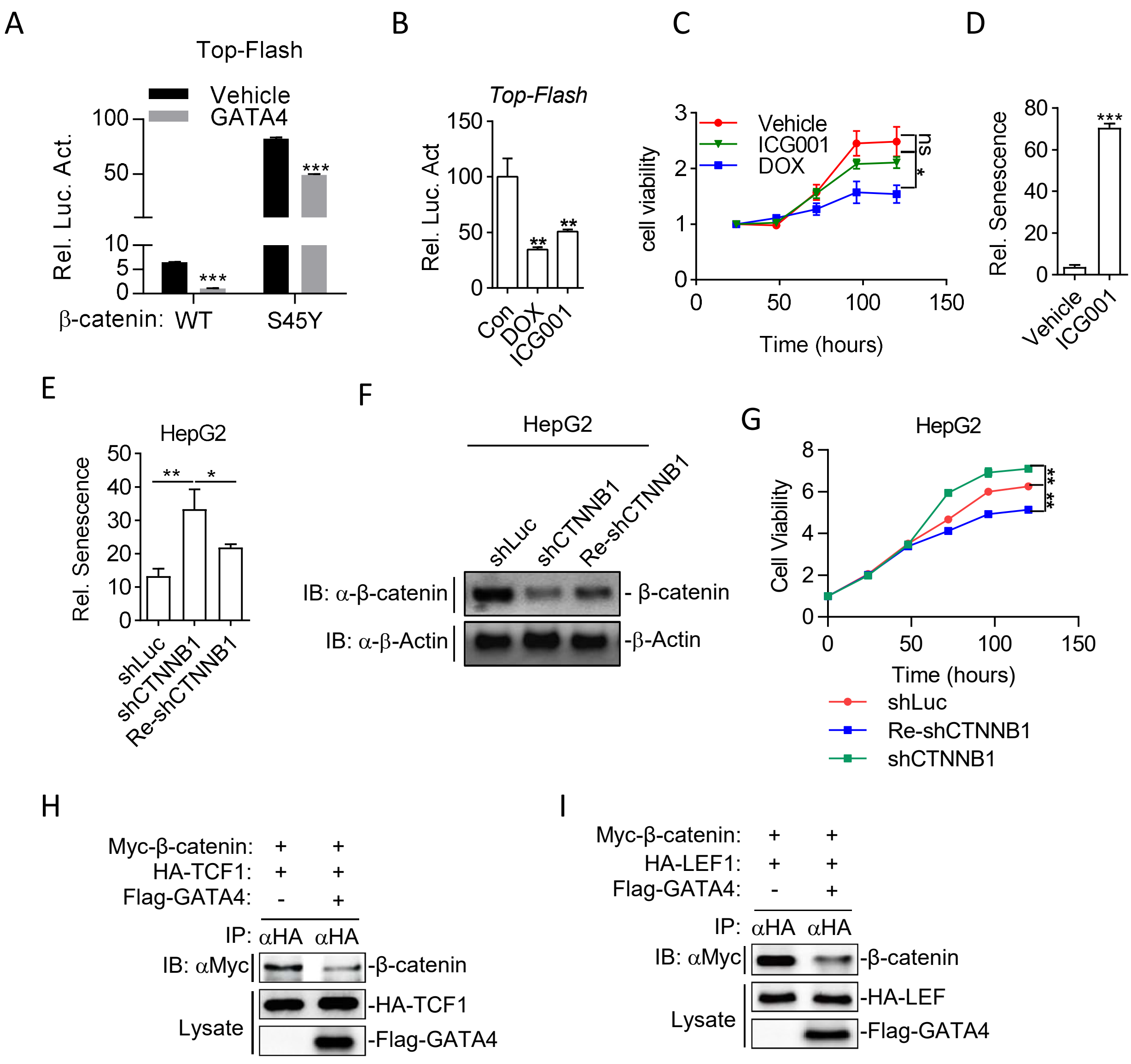

**Figure S4.** GATA4 inhibited transcriptional activity of β-catenin through blocking its recruitment of LEF/TCF1.

**(A)** GATA4 inhibited β-catenin activation. HEK 293 cells ($2\times10^5$) were transfected with the 0.2 μg *Top-Flash* reporter plasmid, 0.3 μg GATA4 expression or its vehicle plasmid with0.3 μg β-catenin (wide type or S45Y mutation ) expression plasmid, followed by monitoring luciferase 24 hours later. **(B)** GATA4 inhibited *TOP-flash* luciferase reporter assay in HepG2i cells. HepG2i cells ($1\times10^5$) were transfected with *Top-flash* reporter plasmid (0.1 μg), followed by treatment with or without DOX (1 μg/mL) or ICG001 (0.1 μg/mL) for 48 hours before luciferase assays were performed. **(C)** GATA4 and ICG001 inhibited cell viability of HepG2i cells. HepG2i cells ($1\times10^3$) were treated with or without DOX (1 μg/mL) or ICG001 (0.1 μg/mL) for 48 hours. Cell viability was detected by CCK-8 assay kit for indicated time points. **(D & E)** ICG001 **(D)** and shCTNNB1 **(E)** induced senescence of liver cancer cells. Statistics of senescence β-galactosidase staining positive cells of Figure 4D. **(F)** Effects of CTNNB1-shRNA plasmids on endogenous β-catenin. Whole-cell lysates from HepG2-Teton-shCTNNB1 cells were analyzed by immunoblots with the indicated antibodies. **(G)** shCTNNB1 inhibited HepG2 cells growth. HepG2 cells engineered for DOX-inducible expression of shCTNNB1 ($1\times10^3$) were treated with or without DOX (1 μg/mL) for 48 hours. Cell viability was then assayed with CCK-8 reagents for indicated time points. **(H & I)** GATA4 inhibited the interaction between β-Catenin and TCF1 (F) or LEF1 (G). The HEK293 cells ($2\times10^6$) were transfected with the indicated plasmids (2.5 μg each). Coimmunoprecipitation and immunoblot were performed with the indicated antibodies.

Data are representative of three independent experiments, and were analyzed by unpaired t-test. Error bars denote SD. *P < 0.05; **P < 0.01; ***P < 0.001

# Lu et al., Figure S5

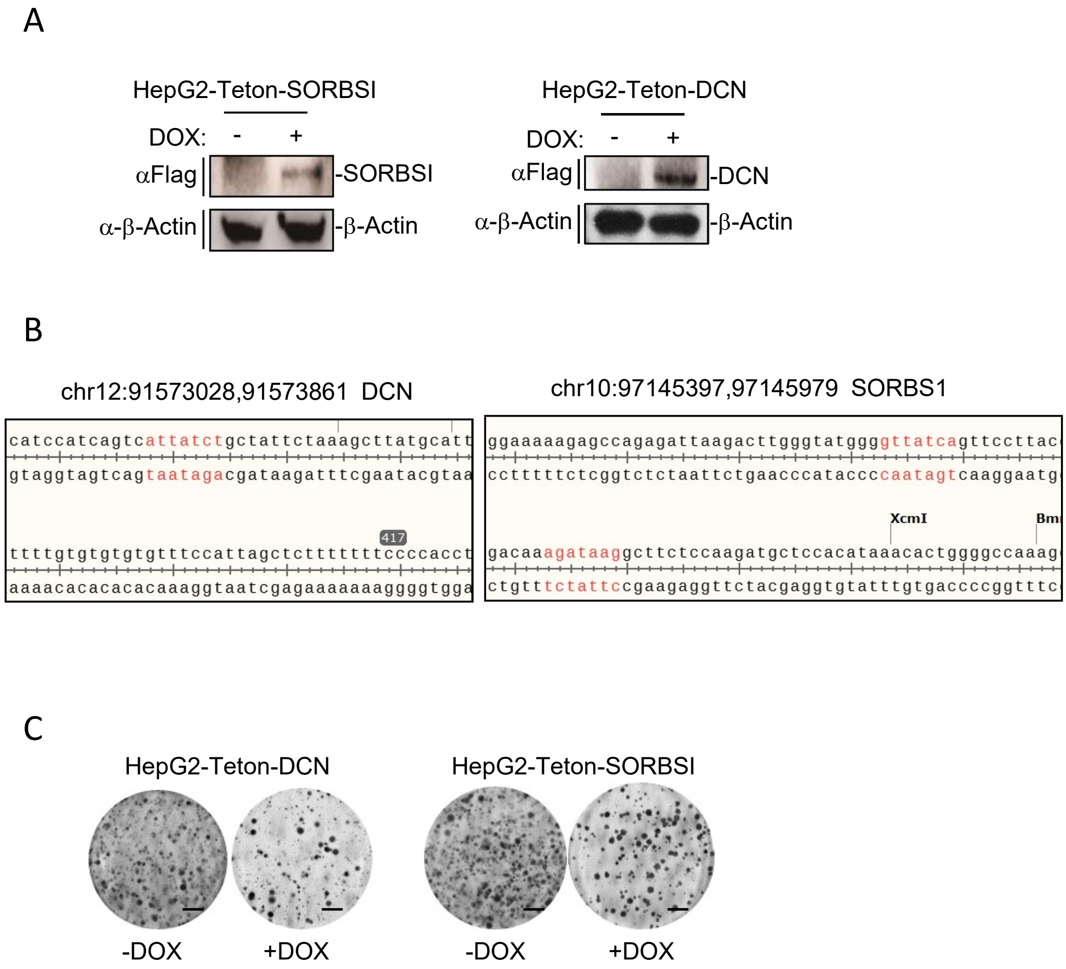


D

| | **high** | **low** |
|---|---|---|
| **mutation** | 24 | 64 |
| **nonmut** | 132 | 137 |
| **mutation rate** | **15.38%** | **31.84%** |

**Figure S5.** SORBSI and DCN inhibited colony forming ability of HepG2.

**(A )** HepG2-Teton-SORBSI cells (2×$10^6$) and Huh7-Teton-DCN cells (2×$10^6$) were left untreated or treated with DOX for 48 hours. Cell lysates were analyzed by immunoblots with the indicated antibodies. **(B)** GATA4 binding consensus sequence is analyzed on the DNA sequence immunoprecipitated from GATA4 expressing HepG2 cells by β-catenin antibody. **(C)** DCN and SORBSI inhibited colony forming of HepG2 cells. Representative images of colony-forming assay of HepG2 cells. (D) Frequency of CTNNB1 mutation in GATA4-high versus GATA4-low patients. We analysed TCGA Liver Cancer (LIHC) data of Frequency of CTNNB1 mutation in GATA4-high versus GATA4-low patients.

Lu et al., Figure S6

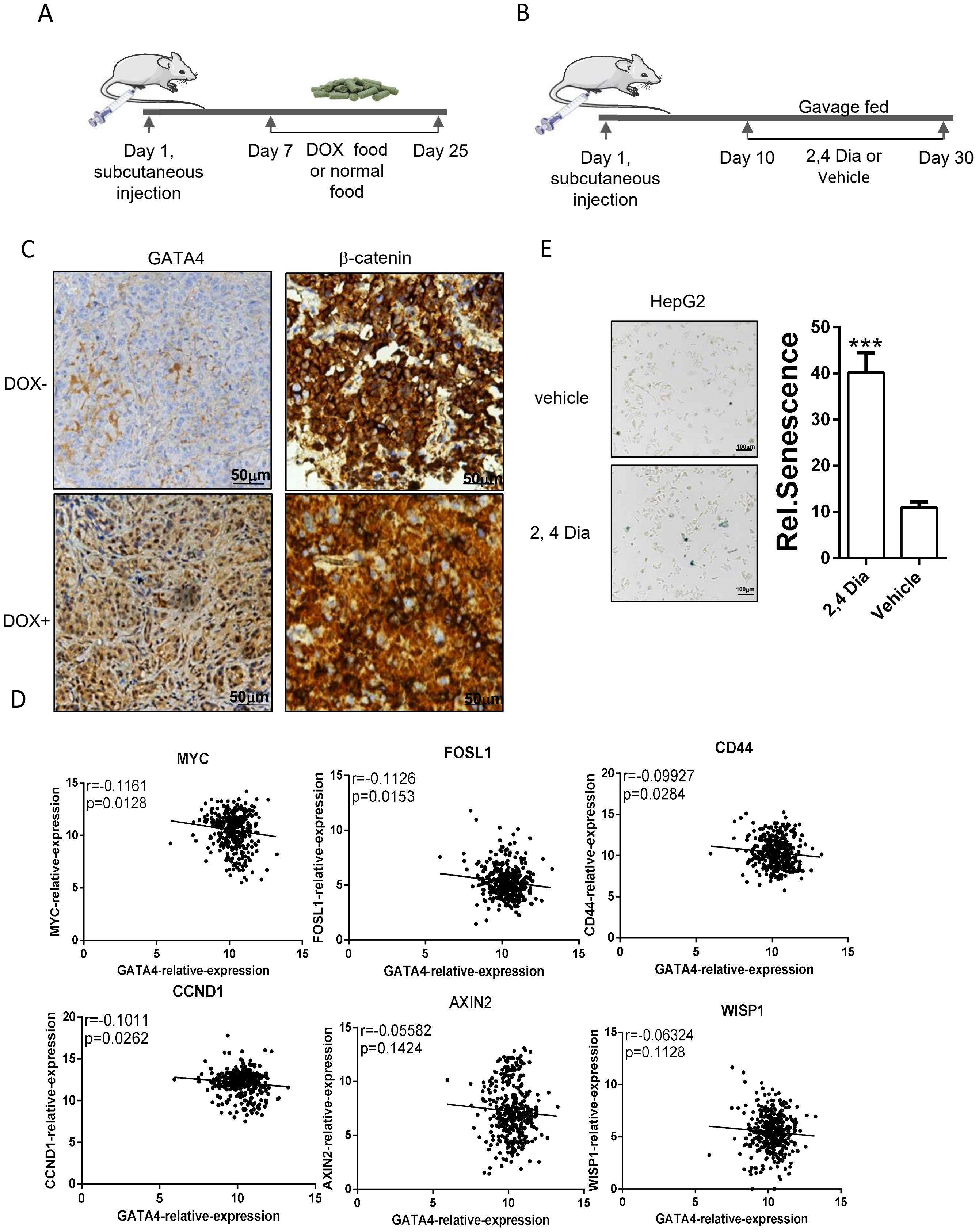

**Figure S6.** Schematics for mice experiments.

**(A)** Schematics for the DOX treatments of nude mice. $5 \times 10^6$ HepG2i cells were inoculated into subcutaneous space on the nude mice. Mice were fed with DOX containing diet or control diet when the tumor volume reached 90 $mm^3$. **(B)** Schematics for 2,4 Dia treatment of nude mice. $5 \times 10^6$ HepG2i cells were inoculated into subcutaneous space on 6-weeks-old male BALB/c nude mice. These mice were administered with 2,4 Dia or vehicle through oral gavage every two days. Body weight was recorded every 5 days and tumor growth was recorded every 3 days.**(C)** The tumor nodules were subjected to IHC staining with GATA4 and β-Catenin (Anti-GATA4 antibody ab61767， β-catenin (D10A8) XP ® Rabbit mAb ) antibody (scale bars:50 μm).**(D)** The correlation between GATA4 and Wnt downstream genes expression was analyzed in TCGA Liver Hepatocellular Carcinoma-(Primary Tumor). **(E) Left:** Senescence-associated β-galactosidase staining (Beyotime Biotechnology ， Senescence β-Galactosidase Staining Kit) of 2,4 Dia and Vehicle treated HepG2 cells. **Right:** statistics of the positive percentage of senescence cells.

**Supplementary experimental procedures:**

## 2.1 Silver staining and Mass spectrometry assays

Silver staining was performed as Silver staining Rapid silver staining kit's description. Cutting the target strip or dividing the strip into several components (strip width is about 2mm) and placed them in 500 μl EP tube. Then experiments were performed according to the protocol of In-gel digestion as described in In-gel digestion for mass spectrometric characterization of proteins and proteomes, Nature Protocols volume 1, pages 2856–2860 (2006), product of In-gel digestion was taken into the Mass spectrometry assays. Mass spectrometry assays was performed on ABI 5600 Triple TOF.

## 2.2 Transfection and Top-Flash Assay

HEK293 cells were transfected by lipofectamine 3,000. Empty control plasmid was added to ensure that each transfection receives the same amount of total DNA. To normalize for transfection efficiency, 0.1 μg of pRL-TK (Renilla luciferase) reporter plasmid was added to each transfection. After 24 hours transfection, ICG001 (1 μg/mL) and DOX (1 μg/ml) were added for 48 hours. Luciferase assays were performed using a dual-specific luciferase assay kit (PROMEGA).

## 2.3 Chromatin immunoprecipitation (ChIP) PCR

HepG2i cells were seeded in eight 150 mm plates, when the cells reached a confluence of 30%~40%, cells received DOX treatment. After 3~4 days of culture, the cells were crosslinked with 1% formaldehyde (final concentration) and sonicated using the following parameters: 5s on, 10s off, 15 cycles at the 25% set power (VCX500, SONICS, CT, USA). Immunoprecipitation was performed with 5 μg of GATA4 antibody or 5 μg of β-catenin antibody, and the immune complexes were absorbed with protein A/G beads. Finally, the eluted DNA was resolved in dd$H_2O$ and processed for further PCR.

## 2.4 Fluorescent confocal microscopy

HepG2 cells were transfected with the indicated plasmids by lipofectamine 3,000. At 24 hours after transfection, the cells were fixed with 4% paraformaldehyde for 10 min

at room temperature. The cells were observed with a Zeiss LSM700 confocal microscope under a ×60 oil objective.

**2.5 GST pull down**

Transfect plasmid PEGX-catenin and PEGX-GATA4 into BL21 *E.coli* and GST pull down assay performed using GST Pull-down standard protocol of Cold Spring Harbor Laboratory Press.

**2.6 Cell cycle Synchronization**

Plate cells at 20-30% confluence in a 10 cm culture dish ($2 \times 10^6$-$3 \times 10^6$ cells per dish) containing 10 ml of DMEM supplemented with 10% Fetal Bovine Serum (FBS). Incubate cells at 37 °C overnight. Add thymidine to a final concentration of 2 mM. Culture cells in a tissue culture incubator at 37 °C for 18 hours. Remove thymidine by washing cells through addition of 10 ml pre-warmed 1x PBS and discard PBS. Add 10 ml of pre-warmed fresh medium and incubate for 9 hours in a tissue culture incubator at 37 °C. Add second round of thymidine to a final concentration of 2 mM. Culture cells at the tissue culture incubator for another 18 hours at 37 °C. Release cells by washing with pre-warmed 1× PBS and incubating cells in pre-warmed fresh media. Cells are collected at 12hours for analysis.

**2.7 Histopathological findings**

Xenograft tumor and liver tissue samples were fixed in 10% neutral buffered formalin, dehydrated, and embedded in paraffin. Samples were subsequently sectioned at 5 µm thickness and stained with hematoxylin and eosin (H&E) for histopathology.

**2.8 IHC Staning**

IHC staning utilized IHC Protocal-1 of Bond max (Leica) and Hot antigen repair applied HIER 25 min with ER2 Protocal of Bond max (Leica).

**2.9 Statistical analysis**

The data are presented as means ± SD of three independent determinations. Statistical analyses were carried out by Student's t-test. $p<0.05$ and $p<0.01$ were considered to be significant.
